# Enhanced 3D Gaussian Splatting for Real-Scene Reconstruction via Depth Priors, Adaptive Densification, and Denoising

**DOI:** 10.3390/s25226999

**Published:** 2025-11-16

**Authors:** Haixing Shang, Mengyu Chen, Kenan Feng, Shiyuan Li, Zhiyuan Zhang, Songhua Xu, Chaofeng Ren, Jiangbo Xi

**Affiliations:** 1 College of Geological Engineering and Geomatics, Chang’an University, Xi’an 710054, China; shang_hx@nwh.cn (H.S.); li_shiyuan@chd.edu.cn (S.L.); 2024126052@chd.edu.cn (Z.Z.); 2024226016@chd.edu.cn (S.X.); rencf@chd.edu.cn (C.R.); xijiangbo@chd.edu.cn (J.X.); 2 Northwest Engineering Corporation Limited, Power China Group, Xi’an 710065, China; 3 Xi’an Key Laboratory of Clean Energy Digital Technology, Xi’an 710065, China

**Keywords:** gaussian splatting, 3D modeling, depth estimation, gradient densification

## Abstract

The application prospects of photorealistic 3D reconstruction are broad in smart cities, cultural heritage preservation, and related domains. However, existing methods face persistent challenges in balancing reconstruction accuracy, computational efficiency, and robustness, particularly in complex scenes characterized by reflective surfaces, vegetation, sparse viewpoints, or large-scale structures. In this study, an enhanced 3D Gaussian Splatting (3DGS) framework that integrates three key innovations is proposed: (i) a depth-aware regularization module that leverages metric depth priors from the pre-trained Depth-Anything V2 model, enabling geometrically informed optimization through a dynamically weighted hybrid loss; (ii) a gradient-driven adaptive densification mechanism that triggers Gaussian adjustments based on local gradient saliency, reducing redundant computation; and (iii) a neighborhood density-based floating artifact detection method that filters outliers using spatial distribution and opacity thresholds. Extensive evaluations are conducted across four diverse datasets—ranging from architectures, urban scenes, natural landscapes with water bodies, and long-range linear infrastructures. Our method achieves state-of-the-art performance in both reconstruction quality and efficiency, attaining a PSNR of 34.15 dB and SSIM of 0.9382 on medium-sized scenes, with real-time rendering speeds exceeding 170 FPS at a resolution of 1600 × 900. It demonstrates superior generalization on challenging materials such as water and foliage, while exhibiting reduced overfitting compared to baseline approaches. Ablation studies confirm the critical contributions of depth regularization and gradient-sensitive adaptation, with the latter improving training efficiency by 38% over depth supervision alone. Furthermore, we analyze the impact of input resolution and depth model selection, revealing non-trivial trade-offs between quantitative metrics and visual fidelity. While aggressive downsampling inflates PSNR and SSIM, it leads to loss of high-frequency detail; we identify 1/4–1/2 resolution scaling as an optimal balance for practical deployment. Among depth models, Vitb achieves the best reconstruction stability. Despite these advances, memory consumption remains a challenge in large-scale scenarios. Future work will focus on lightweight model design, efficient point cloud preprocessing, and dynamic memory management to enhance scalability for industrial applications.

## 1. Introduction

Real-scene 3D modeling technology, by inputting multi-view images and processing them through photogrammetric algorithms, enables the generation of high-precision, high-fidelity 3D models of real-world scenes within computers. The standard technical workflow begins with planning flight paths to acquire multi-angle images of the target objects for Unmanned Aerial Vehicles (UAVs) equipped with remote sensing sensors. Subsequently, a sparse reconstruction is performed using Structure from Motion (SfM) [1], followed by a dense reconstruction step utilizing Multi-View Stereo (MVS) [2]. Finally, the 3D model is generated through texture mapping. This technology is not only extensively applied in surveying and mapping but has also become a core enabling tool in fields such as urban planning, cultural heritage preservation, and autonomous driving, owing to its efficiency, accuracy, and strong applicability. However, its modeling efficiency significantly decreases, and precision becomes difficult to guarantee when processing complex scenes and dynamic scenes.

Neural Radiance Fields (NeRFs) [3] leverage deep learning to infer the geometry and texture characteristics of a target scene from input multi-view images, outputting a three-dimensional radiance field. This technique employs continuous functions to represent the density and color attributes of points within 3D space, replicating the interaction of light rays with object surfaces in the real world. Consequently, it enables the rendering of projected images of the 3D model from arbitrary viewpoints and distances. However, reconstructing continuous 3D scenes via neural networks incurs significant costs: (i) the inability to perform post-reconstruction editing hinders its applicability in domains requiring frequent scene modifications, such as game asset creation and engineering supervision; (ii) due to its reliance on extensive multilayer perceptrons (MLPs) and dense ray sampling, training times for large-scale scenes often extend to tens of hours, while rendering speeds are limited to merely a few frames per second (FPS), increasingly failing to meet the efficiency demands of various industries.

In August 2023, Bernhard Kerbl et al. from the I3S Laboratory, Université Côte d’Azur, France, introduced 3D Gaussian Splatting (3DGS) [4], a novel 3D scene representation method based on 3D Gaussian functions and rasterization-based rendering. This technique offers three primary advantages: (i) the introduction of anisotropic 3D Gaussian ellipsoids as a flexible scene representation primitive [5]; (ii) efficient rasterization achieved through projection onto 2D image planes; (iii) high-quality, unstructured representation of the radiance field via continuous optimization of anisotropic covariance, spherical harmonic (SH) coefficients [6], opacity, and adaptive density control. Consequently, 3DGS achieves high-quality real-time rendering exceeding 30 FPS at 1080p resolution without requiring extensive training time, while maintaining exceptional reconstruction fidelity and visual quality. Furthermore, the “explicit” geometric nature of 3DGS grants the generated models inherent editability [7].

Despite the high-quality reconstruction capabilities of 3DGS, it faces significant challenges in weak-texture regions [8]. Traditional SfM techniques struggle to generate sufficient initialization point clouds on weak-texture surfaces, leading to subsequent geometric inaccuracies during Gaussian optimization. To address this, GaussianPro proposed a progressive propagation strategy. This method drives the adaptive densification of the 3D Gaussian point cloud through an iterative update mechanism that jointly enforces normal consistency constraints and planar priors. Compared to the original split-and-clone strategy, this approach elevates the PSNR metric by 1.15 dB on the “Waymo” street-view dataset [9], significantly enhancing geometric completeness and rendering fidelity in weak-texture areas.

Concurrently, to mitigate the storage and computational burden arising from the massive number of Gaussians in 3DGS, Compact3D introduced a vector quantization framework incorporating run-length encoding and zero-opacity regularization. By suppressing invalid Gaussian distributions, this strategy achieves a 40–50× model compression ratio while boosting rendering efficiency by 2–3× [10]. Similarly, LightGaussian constructed a multi-stage compression pipeline. It filters Gaussians based on global saliency and employs VecTree hybrid quantization for hierarchical compression of geometric features. On datasets like “Mip-NeRF 360” and “Tank & Temple,” it achieves an average compression rate exceeding 15×, concurrently increasing rendering frame rates from 139 FPS to 215 FPS. This breakthrough overcomes VRAM limitations while preserving visually lossless quality [11].

Beyond compression, recent work has also explored improving robustness in sparse-view scenarios and integrating depth estimation with 3DGS. D^2^GS [12] improves robustness under sparse-view conditions by introducing a Depth-and-Density Guided Dropout to suppress overfitting near the camera and a Distance-Aware Fidelity Enhancement module to address underfitting in distant regions, validated via a novel stability metric. In contrast, DepthSplat [13] integrates monocular depth estimation with Gaussian splatting, leveraging pretrained depth features for high-quality feed-forward reconstructions while enabling unsupervised pre-training of depth models on large-scale datasets. Their bidirectional synergy achieves state-of-the-art performance in depth estimation and novel view synthesis. While D^2^GS emphasizes geometric stability through localized optimization strategies, DepthSplat prioritizes task integration by bridging depth estimation and rendering pipelines. These approaches represent distinct yet complementary directions for improving 3DGS: the former targets hardware-efficient sparse-view modeling, while the latter advances cross-task generalization via joint training. Both frameworks, however, share the common goal of reducing dependency on dense input views, thereby expanding 3DGS’s applicability to real-world scenarios with limited camera coverage.

For dynamic scene modeling, 4DGS re-engineered the 3DGS paradigm by decoupling 3D space and the temporal dimension into a 4D continuous field. It utilizes multi-resolution neural voxel decomposition to build spatio-temporal feature correlations and employs lightweight MLPs to decode deformation parameters, enabling continuous motion and deformation prediction of Gaussian point clouds along the time axis [14]. This method describes an object’s spatial distribution and geometry at each time instance using a set of 3D Gaussians, while temporal variations are captured via 4D neural voxels. This representation efficiently captures geometric changes and motion information within dynamic scenes, allowing continuous temporal evolution and enabling highly efficient rendering. This hybrid explicit–implicit representation resolves the traditional trade-off between efficiency and accuracy in dynamic modeling. Leveraging its rasterization-friendly pipeline, it achieves real-time rendering at 82 FPS (800 × 800 resolution) on an RTX 3090 GPU, accelerating training by 5× compared to dynamic NeRF methods, thus effectively balancing the efficiency-precision dilemma [15].

Beyond dynamic scenes, the complexity of city-scale scenes imposes higher demands on 3D technology. VastGaussian and CityGaussian, as breakthrough techniques for urban-scale rendering, address the challenges of efficiency and precision through distinct approaches. VastGaussian emphasizes spatial coherence and appearance consistency. It proposes a progressive spatial-aware partitioning strategy, dividing the city-scale scene into spatial units based on camera projection and employing decoupled appearance modeling. This achieves 82 FPS real-time rendering at 1080p resolution on datasets like “Mill-19,” and the total Gaussian count for the merged scene can exceed single-GPU VRAM limits [16], significantly enhancing reconstruction detail density. CityGaussian focuses on dynamic computational resource allocation and geometric fidelity assurance. It optimizes multi-scale rendering efficiency by constructing a joint framework for tile-based parallel training and dynamic Level-of-Detail (LoD). This enables real-time navigation at 53.7 FPS over a 2.5 km^2^ urban area, representing a 2.5× improvement over baseline methods [17].

These advancements lay a solid foundation for the technological transformation and application of 3DGS. Gaussian-SLAM [18], a representative system, is a real-time dense visual SLAM (Simultaneous Localization and Mapping) method based on 3D Gaussian representation, designed to tackle high-precision localization and scene reconstruction in complex dynamic environments. This system innovatively adopts 3D Gaussians as the fundamental scene representation. By deeply integrating Gaussian parameter optimization with the SLAM pipeline via differentiable rendering, it achieves sub-millimeter reconstruction accuracy and operates at over 30 FPS. Furthermore, its Gaussian optimizer, based on physical collision constraints and a dynamic Gaussian masking mechanism, effectively mitigates localization drift in weak-texture areas and ensures stable operation amidst dynamic interferences like pedestrians and vehicles. Experiments demonstrate approximately 37% higher localization accuracy compared to ORB-SLAM3 on benchmark datasets like “TUM RGB-D.” It surpasses NeRF-based methods in reconstruction quality while reducing computational resource consumption to 1/5. This technology provides a novel solution for autonomous driving and augmented reality, which demand real-time high-precision environmental perception, exhibiting significant advantages particularly in outdoor open scenes and indoor dynamic environments.

In summary, current research on 3DGS demonstrates a trend moving from fundamental algorithm optimization towards in-depth exploration of complex application scenarios. Breakthroughs in city-scale scene modeling and SLAM systems mark significant strides towards engineering deployment [19,20]. Nevertheless, balancing the competing demands of real-time rendering, computational resource constraints, and operational robustness under dynamic environmental complexities remains an unresolved challenge. Future research necessitates continued exploration in directions such as network architecture lightweighting, multi-modal data fusion, and adaptive scene understanding to promote the universal adoption of 3D reconstruction technology in industrial-grade applications.

This study focuses on the optimization and refinement of 3DGS technology, aiming to address its limitations in complex scene reconstruction, including unstable geometric optimization, inefficient adaptive density control, and initialization noise interference. Therefore, we propose an enhanced 3DGS framework with a gradient-aware dynamic adjustment that introduces three key innovations: (i) a depth-aware regularization module that leverages metric depth priors from the pre-trained Depth-Anything V2 model, enabling geometrically informed optimization through a dynamically weighted hybrid loss; (ii) a gradient-driven adaptive densification mechanism that triggers Gaussian adjustments based on local gradient saliency, reducing redundant computation; and (iii) a neighborhood density-based floating artifact detection method that filters outliers using spatial distribution and opacity thresholds. The research encompasses algorithmic innovation, theoretical validation, and engineering practice, systematically advancing the performance boundaries of 3DGS in real-scene 3D modeling.

The rest of the paper is organized as follows: Section 2 introduces the technical workflow of 3DGS and its improved methods. Section 3 presents the experimental results and discussions. Section 4 outlines the conclusions of the research method employed in this study.

## 2. Methodology

### 2.1. Principles of 3D Gaussian Splatting

**Figure 1:**
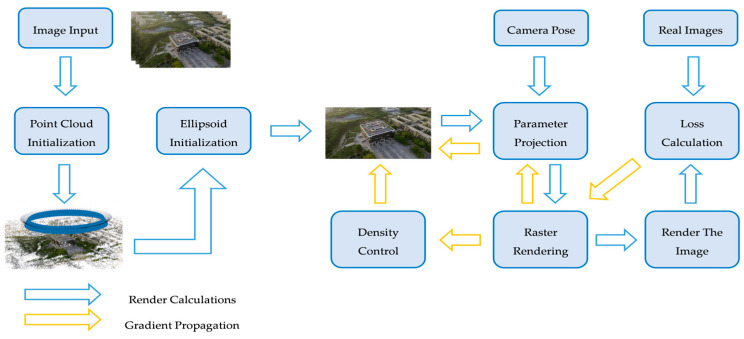
Gaussian Splatting Technical Workflow.

The core of 3DGS technology lies in its use of a multitude of 3D Gaussian ellipsoids to achieve a compact representation of the scene. Subsequently, an optimized rendering pipeline projects the 3D information onto the 2D image plane, ultimately generating photorealistic visual effects. The entire technical workflow of 3DGS primarily revolves around two key aspects: error computation and gradient propagation. Its operational mechanism resembles optimization processes common in the machine learning domain, progressively enhancing the accuracy of scene representation and the quality of rendered output through iterative refinement. This process can be summarized into the following key stages (Figure 1):

#### 2.1.1. Point Cloud Initialization

After acquiring a sequence of 2D images of the target scene using drones, mobile devices, or professional camera systems, 3DGS employs SfM technology to construct the scene’s geometric structure. By extracting image feature points and performing cross-view matching, combined with bundle adjustment optimization algorithms, the system simultaneously solves the spatial pose parameters of the cameras and reconstructs a sparse 3D point cloud of the scene via the open-source library COLMAP [21]. Within the 3DGS framework, this sparse point cloud serves as the core geometric scaffold. Each point is transformed into the initial carrier of a 3D Gaussian ellipsoid: its positional parameters are directly inherited from the point cloud coordinates, while the ellipsoid’s rotation and scaling attributes are parametrically expressed through the decomposition of its covariance matrix. 

#### 2.1.2. Gaussian Ellipsoid Initialization

Gaussian ellipsoid initialization utilizes the spatial positions of the point cloud as its structural skeleton, endowing them with dynamic expressive capabilities for geometric forms. In one-dimensional space, a Gaussian distribution determines its central position through the mean μ and the variance $\sigma^{2}$. In three-dimensional space, Gaussian distributions jointly characterize the geometric properties of ellipsoids through a mean vector and a covariance matrix. In the one-dimensional case, the probability density function of a Gaussian distribution is given by:

(1) $$f(x)=\frac{1}{\sigma \sqrt{2\pi }}e^{-\frac{1}{2}{\left(\frac{x-\mu }{\sigma }\right)}^{2}}$$

where the mean μ determines the central position of the bell-shaped curve, and the variance $\sigma^{2}$ controls its shape.

When extended to 3D space, this characteristic evolves into a Gaussian ellipsoid model centered at the mean vector μ with the covariance matrix Σ as its shape-controlling parameter. The probability density function is given by:

(2) $$f(x)=\frac{1}{\sqrt{{\left(2\pi \right)}^{3}|\Sigma |}}e^{-\frac{1}{2}{\left(x-\mu \right)}^{T}\Sigma^{-1}(x-\mu )}$$

Since the density of a Gaussian distribution is highest at its center, Equation (2) can be simplified to:

(3) $$G(x)=e^{-\frac{1}{2}{\left(x-\mu \right)}^{T}\Sigma^{-1}(x-\mu )}$$

The covariance matrix Σ determines the scaling, compression, and rotational state of the ellipsoid in 3D space. Through matrix decomposition, it can be factorized into a rotation matrix R and a scaling matrix S as follows:

(4) $$\Sigma =RSS^{T}R^{T}$$

The spatial positional data of the point cloud can be derived through the rotation matrix R and scaling matrix S.

#### 2.1.3. Projection and Rasterization Rendering

Upon acquiring the 3D Gaussian ellipsoids, they must be mapped from 3D space to a designated 2D plane based on the camera viewpoint. This process is termed “splatting” (also referred to as “splat rendering”), named for its resemblance to the imprint left by a snowball impacting a wall. Essentially, when a 3D ellipsoid undergoes nonlinear projection onto a 2D plane, the density at its center contributes most significantly to pixel color, while contributions from peripheral regions attenuate with distance [22,23,24]. Specifically, the view transformation matrix W first transforms the ellipsoid from the world coordinate system to the camera coordinate system, where its covariance matrix Σ still characterizes its geometric distribution in 3D space. Since perspective projection is inherently a nonlinear operation, directly manipulating transformations of the covariance matrix would complicate computations. Therefore, a Jacobian matrix J is introduced to locally linearize the projection process. This enables the mapping of the 3D ellipsoid’s covariance matrix onto the projection plane to be formulated as a linear algebraic operation, ultimately computed using the following equation:

(5) $$\Sigma^{\prime }=JW\Sigma W^{T}J^{T}$$

To achieve high-speed rendering, 3DGS further employs a tile-based rasterizer. Before rendering commences, the target image is partitioned into 16 × 16 square tiles. A spatial filtering mechanism extracts Gaussian ellipsoids within each tile’s view frustum that meet two criteria: (i) confidence metrics exceed a predefined threshold; (ii) they reside within the current viewpoint’s visible range. Subsequently, the selected ellipsoids are strictly sorted by spatial depth to ensure correct occlusion handling during rendering. During the parallel computation phase, each tile instance is assigned an independent thread. These threads execute reverse splatting rendering based on the sorted ellipsoid sequence, beginning with primitives closest to the imaging plane. Spatial projection proceeds sequentially, with multi-layer feature superposition forming the basis for rasterized data. When the cumulative opacity within a pixel region reaches full saturation, the corresponding tile thread automatically terminates computation. This enables dynamic optimization of rendering resources.

#### 2.1.4. Loss and Density Control

In the optimization process, the design of the loss function and the dynamic control of the Gaussian primitive density are key components for improving the quality of scene representation. The loss function adopts a multi-objective weighted form, defined as:

(6) $$L=(1-\lambda )L_{1}+\lambda L_{\mathrm{SSIM}}$$

where the $L_{1}$ denotes the pixel-wise *L*_1_ loss, which enforces global color consistency in the optimization process by comparing differences between rendered and ground-truth images on a per-pixel basis. The $L_{\mathrm{SSIM}}$ represents the structural similarity loss, preserving high-frequency details and reducing blurring artifacts through measurements of luminance, contrast, and structural similarity within local image windows, λ serves as the coefficient balancing the weights between these two loss terms.

To achieve more precise scene representation, upon completion of the initial warm-up phase (i.e., after exceeding 500 iterations), Gaussian densification is triggered every 100 iterations while ellipsoids with opacity below a predefined threshold are pruned. Through this density control mechanism, the Gaussian count progressively scales from an initially sparse point cloud to millions of primitives, ultimately forming a compact yet unstructured high-fidelity scene representation.

### 2.2. Refinements and Optimizations of Key Procedures

#### 2.2.1. Dynamic Weighted Supervision Method Based on Depth Prediction Network

To address the challenge of unstable geometric optimization in 3D reconstruction, this study proposes a dynamic weighted supervision strategy based on depth prediction networks. This approach integrates depth priors extracted from pre-trained depth networks [25] to formulate a depth-aware hybrid loss function. Simultaneously, it dynamically adjusts constraint strengths during optimization to effectively balance the relationship between depth guidance and photometric consistency. The core workflow comprises three critical stages: depth prediction, depth rendering, and dynamic loss construction, collectively forming a comprehensive depth regularization scheme:

1. Depth Prediction for Constructing Geometric Priors

First, dense depth prediction is performed on input images using the Depth-Anything V2 model [26]. Jointly developed by The University of Hong Kong and ByteDance, this model employs a synthetic data-driven large-scale pre-training paradigm. It generates high-precision pseudo-labels through a teacher model based on the DINOv2-Giant architecture and incorporates gradient-matching loss to optimize fine-grained features. Notably, Depth-Anything V2 is explicitly designed to produce scale-aware metric depth across diverse scenes, enabling direct use as geometric priors without per-scene scaling or calibration. In contrast, many widely used monocular depth models (e.g., MiDaS, DPT) primarily predict relative depth rankings or normalized depth maps that preserve local structure but lack global scale consistency. This capability makes Depth-Anything V2 particularly suitable for 3D reconstruction tasks requiring reliable geometric initialization. The model offers four scalable versions ranging from 25 million to 1.3 billion parameters. Compared to traditional methods reliant on COLMAP sparse point clouds, it directly outputs depth maps aligned with input resolution via cross-domain scale consistency modeling, significantly reducing computational complexity while enhancing the reliability of depth priors.

For the input image $I\in [0,\;1{]}^{H\times W\times 3}$, its depth prediction process can be expressed as:

(7) $$D_{\mathrm{pred}}=F_{\theta }(I)$$

where $F_{\theta }$ represents the parameterized function of the depth network. Currently, the available models include three types: Vits, Vitl, and Vitb.

2. Depth Rendering

We extend the 3DGS rendering pipeline with a depth rendering branch to support depth optimization. This is mainly based on the transparency blending mechanism of Gaussian ellipsoids, where the pixel depth value $D_{\mathrm{render}}$ is calculated through visibility sorting and weighted accumulation:

(8) $$D_{\mathrm{render}}=\sum_{i\in V(p)}d_{i}\cdot \alpha_{i}\prod_{j=1}^{i-1}(1-\alpha_{j})$$

where V(p) denotes the set of Gaussian ellipsoids covering the pixel p, sorted in ascending order of distance to the camera. Here, $d_{i}$ represents the depth value of the *i*-th Gaussian ellipsoid’s center in the camera coordinate system, while $\alpha_{i}$ and correspond to its opacity and effective coverage derived from the covariance matrix, respectively.

This Equation (8) inherits the blending framework of 3DGS, ensuring strict physical consistency between depth computation and color rendering processes.

3. Dynamic Weighted Depth Loss Function

To coordinate the contributions of depth regularization and color optimization in different training stages, the experiment designs an adaptive weight adjustment mechanism based on exponential space interpolation. The depth loss term is defined as the normalized *L*_1_ distance between the predicted depth and the rendered depth:

(9) $$L_{\mathrm{depth}}=\frac{1}{HW}\sum_{p=1}^{HW}|D_{\mathrm{render}}(p)-D_{\mathrm{pred}}(p)|$$

The depth weighting coefficient λ(t) follows a log-linear interpolation scheme, progressively transitioning from its initial value $\lambda_{0}$ to $\lambda_{T}$ during training:

(10) $$\lambda (t)=\exp((1-t)\ln(\lambda_{0})+t\ln(\lambda_{T}))$$

where the normalized progress parameter t = max(0, min(1, k/K)) is dynamically computed from the current iteration k and total iterations K.

This strategy strengthens depth constraints during early training stages to guide Gaussian ellipsoids toward rapid convergence to a plausible spatial distribution, while progressively attenuating depth supervision in later phases to prevent overfitting. This facilitates a natural transition of optimization focus toward refined reconstruction of color and texture. By integrating the depth loss term, the final loss function becomes:

(11) $$L=(1-\lambda )L_{1}+\lambda L_{\mathrm{SSIM}}+\lambda (t)L_{\mathrm{depth}}$$

The incorporation of depth prediction networks and a dynamic weighting mechanism yields an efficient, adaptive depth regularization framework for few-shot 3DGS.

#### 2.2.2. Gradient-Driven Dynamic Densification Triggering Method

In the conventional 3DGS algorithms, densification serves as the core mechanism for optimizing geometric representation. Its objective is to balance the expression of scene detail and computational efficiency by dynamically adjusting the quantity and distribution of Gaussian ellipsoids. The original densification strategy employs a periodic triggering pattern: after exceeding 500 iterations, cloning and splitting operations are executed every 100 iterations. This process selects Gaussians requiring adjustment based on gradient magnitude thresholds, filters undersized ellipsoids via size thresholds to suppress noise, and prunes ellipsoids with excessively low opacity. Although this regularized operation enhances geometric completeness to some extent, its fixed triggering frequency and static threshold settings may introduce computational redundancy when Gaussian distributions have already converged, resulting in a suboptimal trade-off between reconstruction accuracy and computational efficiency.

To address the aforementioned limitations, this work proposes a gradient-driven adaptive densification trigger mechanism. Its core innovation lies in directly linking geometric adjustments to local gradient saliency, achieving “on-demand optimization” through adaptive control. Specifically, we define g as the average magnitude of positional gradients for currently visible Gaussian primitives—a physical quantity directly reflecting the instability level of geometric structures. The dynamic threshold $G_{d}$ is designed to progressively decay throughout training:

(12) $$G_{d}=G_{0}\cdot \left(1-\frac{k}{K}\right)$$

where $G_{0}$ is the initial threshold, k is the current number of iterations, and K is the total number of iterations.

According to Equation (12), the dynamic threshold $G_{d}$ employs linear decay to simulate the natural gradient descent trend during training: a lenient triggering policy is permitted in early stages to rapidly establish foundational geometry, while progressively stricter thresholds are enforced in later stages to suppress excessive splitting. The densification process, which comprises cloning, splitting, and pruning strategies, is activated only when the real-time gradient satisfies $g\;>\;G_{d}$.

#### 2.2.3. Density-Based Floating Artifacts Detection and Pruning Method

**Figure 2:**
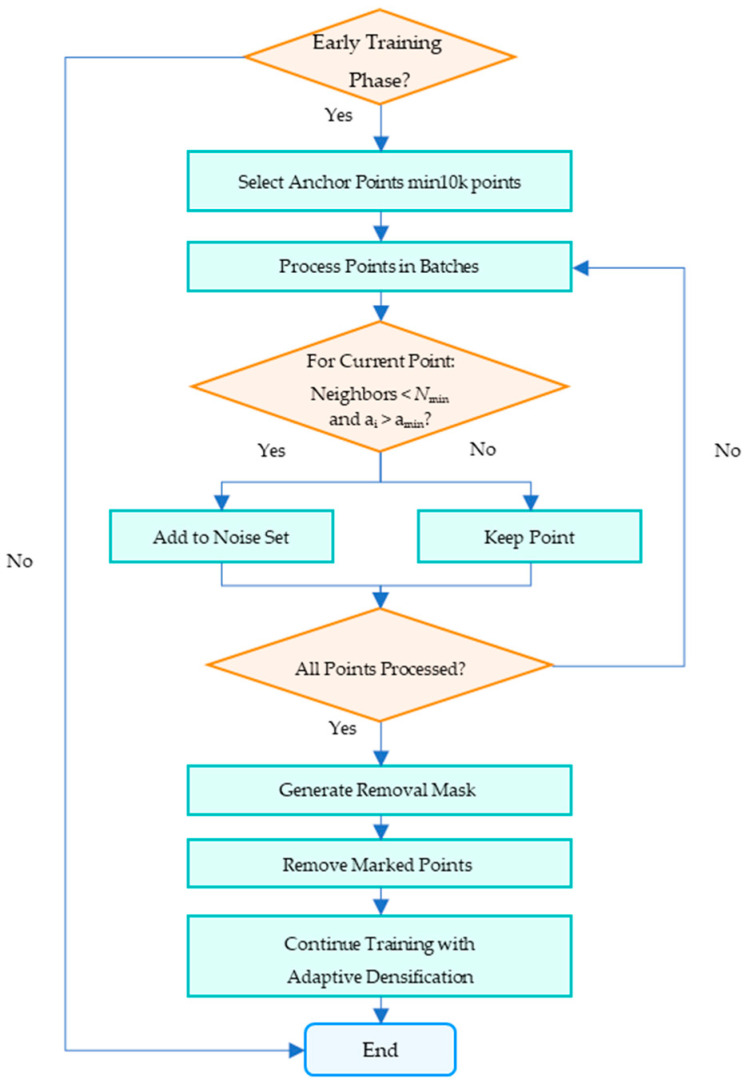
The workflow of the density-based floating artifacts detection and pruning.

In the optimization process of 3DGS, although densification operations can enhance the ability to express scene geometric details, with the dynamic growth in the number of Gaussian ellipsoids, noise in the initial point cloud and floating artifacts generated during optimization [27] will significantly affect reconstruction quality. Such noise points usually manifest as Gaussian primitives with isolated spatial distribution and lack of surrounding topological support, and their abnormally high opacity properties tend to cause local overfitting. To address this, this study proposes a floating noise detection and culling method based on neighborhood density. It eliminates potential interference sources before densification through a pre-filtering strategy to improve the purity of geometric optimization. The core of this method lies in defining the judgment rules for floating noise: for any center point of a Gaussian ellipsoid, if there are fewer than $N_{\min}$ neighboring points within its neighborhood of radius r, and its opacity $\alpha_{i}$ is greater than the preset threshold $\alpha_{\min}$, it is determined as a floating noise point that needs to be culled. The specific detection workflow is as follows:

First, 10,000 points are randomly sampled from the entire 3D point cloud as reference anchors. If the current point cloud contains fewer than 10,000 points, all points are utilized to construct an approximate spatial distribution model. Second, for each candidate point, the algorithm employs a block-wise processing strategy: only Euclidean distances between the candidate and a subset of anchors are computed per iteration, preventing memory overflow from full-data processing. Third, the algorithm restricts final validation exclusively to candidate points with opacity exceeding a preset threshold. All qualifying ellipsoids are then marked with a binary mask to identify target regions for subsequent pruning. Genuine surface points accidentally removed will be naturally replenished through gradient-driven adaptive density control during subsequent densification, establishing a self-correcting closed-loop optimization workflow (Figure 2).

#### 2.2.4. Technical Workflow of the Optimized 3DGS Method

**Figure 3:**
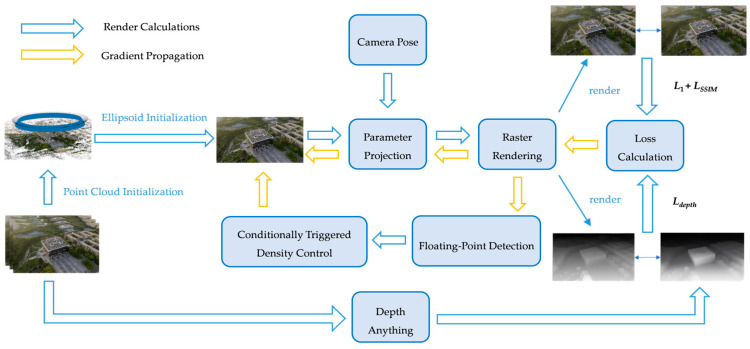
Optimized Gaussian Splatting Technical Workflow.

The optimized 3DGS algorithm workflow addresses the defects of the original method in geometric optimization, dynamic density control, and initialization noise through systematic optimization. The flowchart of the optimized Gaussian Splatting technology is shown in Figure 3:

The workflow starts with inputting multi-view images. First, a sparse point cloud is generated through SfM, and the Depth-Anything V2 depth prediction network is introduced to perform dense depth estimation on the input images, providing geometric priors for subsequent optimization. Meanwhile, by constructing a dynamically weighted hybrid loss function, depth constraints are strengthened in the early stages of training to guide Gaussian ellipsoids to quickly converge to a reasonable spatial distribution, while depth supervision is gradually weakened in the later stages, shifting the focus to refined reconstruction of colors and textures.

Subsequently, a gradient-driven dynamic densification triggering mechanism is adopted to replace the original fixed-cycle density adjustment strategy, dynamically adjusting the Gaussian distribution density by real-time monitoring of local gradient saliency. When the position gradient magnitude of Gaussian ellipsoids exceeds the dynamic threshold that decays with the training process, the system automatically triggers cloning and splitting operations to avoid redundant computations and improve optimization efficiency.

Before the densification operation, the newly added floating noise detection module jointly determines abnormal points based on neighborhood density and opacity thresholds. It screens isolated high-opacity points through spatial distribution statistics and generates a binary mask for culling, thereby optimizing the quality of the initial point cloud. During the optimization process, the rotation, scaling, color (spherical harmonic coefficients), and opacity parameters of Gaussian ellipsoids are continuously updated through backpropagation, and finally, high-quality 3D scenes are generated through block-parallel rasterization rendering.

The refined workflow establishes a closed-loop optimization system encompassing depth-guided initialization, gradient dynamic densification, and pre-densification detection, effectively enhancing the robustness and efficiency of complex scene reconstruction.

### 2.3. Evaluation Metrics

This study employs three types of metrics to systematically evaluate the algorithm performance, covering three dimensions: pixel-level error, structural similarity, and perceptual consistency. The specific definitions and calculation methods are as follows:

#### 2.3.1. Peak Signal-to-Noise Ratio (PSNR)

PSNR reflects the fidelity of the reconstructed results by quantifying the pixel-level difference between the reconstructed image and the real image. Its calculation is based on the Mean Squared Error (MSE), with the formula as follows:

(13) $$\mathrm{MSE}=\frac{1}{H\times W}\sum_{i=1}^{H}\sum_{j=1}^{W}{\left(I_{\mathrm{gt}}(i,j)-I_{\mathrm{pred}}(i,j)\right)}^{2}$$

where H and denote the height and width of the image, while $I_{\mathrm{gt}}$ and $I_{\mathrm{pred}}$ represent the pixel values of the ground-truth and rendered images, respectively.

PSNR further converts MSE into a logarithmic scale [28]:

(14) $$\mathrm{PSNR}=10\cdot \log_{10}\left(\frac{{\mathrm{MAX}}^{2}}{\mathrm{MSE}}\right)$$

where MAX is the maximum range of pixel values, which is 255 for 8-bit images.

A higher PSNR value indicates smaller pixel-level errors between the reconstructed and ground-truth images, though it may fail to capture the human visual system’s sensitivity to high-frequency details.

#### 2.3.2. Structural Similarity Index (SSIM)

SSIM comprehensively evaluates image similarity from three dimensions: Luminance, Contrast, and Structure, with its equation as follows [28]:

(15) $$\mathrm{SSIM}(x,y)=\frac{\left(2\mu_{x}\mu_{y}+C_{1})(2\sigma_{xy}+C_{2}\right)}{\left(\mu_{x}^{2}+\mu_{y}^{2}+C_{1})(\sigma_{x}^{2}+\sigma_{y}^{2}+C_{2}\right)}$$

where $\mu_{x}$ and $\mu_{y}$ are the means of local image windows, $\sigma_{x}$ and $\sigma_{y}$ are the standard deviations, $\sigma_{xy}$ is the covariance, and $C_{1}$ and $C_{2}$ denote stabilization constants that prevent division by zero, conventionally defined as $C_{1}={\left(0.01L_{p}\right)}^{2}$ and $C_{2}={\left(0.03L_{p}\right)}^{2}$ with $L_{p}$ representing the maximum pixel value of the image data.

The value of SSIM ranges from [0, 1], with a value closer to 1 indicating higher structural similarity. It is particularly good at capturing the matching degree of edges and textures.

#### 2.3.3. Learned Perceptual Image Patch Similarity (LPIPS)

LPIPS extracts high-level semantic features of images through a pre-trained deep neural network and calculates the distance in the feature space to simulate differences in human visual perception [29]. The deep neural network selected here is the VGG network, and its calculation method is as follows:

(16) $$\mathrm{LPIPS}(x,y)=\sum_{l}w_{l}\cdot F_{l}(x)-F_{l}{\left(y\right)}_{2}^{2}$$

where $F_{l}$ denotes the feature map of the *l*-th layer.

A lower LPIPS value indicates a higher perceptual similarity between the two images, and it is particularly suitable for evaluating high-frequency details and semantic consistency.

The experiment was fixed at 30,000 iterations, with training results saved and evaluation metrics outputted at the 7000th iteration. Parameters were updated using the Adam optimizer, with learning rates set as follows: 0.0025 for color features, 0.05 for opacity, 0.005 for scaling factors, and 0.001 for rotation parameters. A sampling ratio of 0.01 was adopted during the densification process.

## 3. Experiment Results and Discussion

### 3.1. Study Data

**Figure 4:**
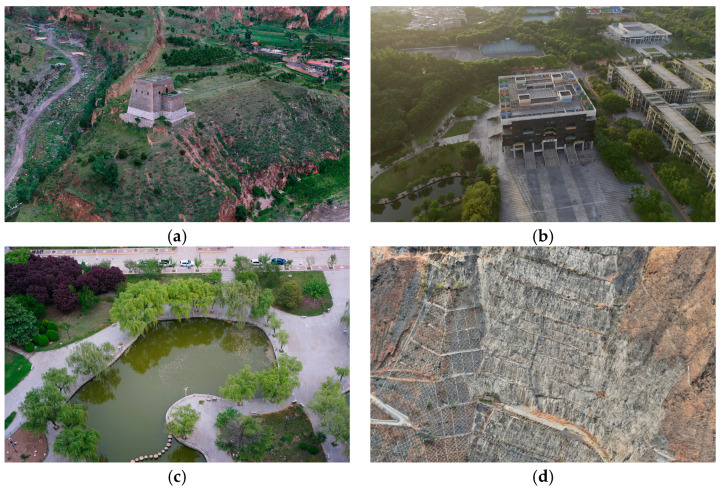
The scenes of the four types of experimental data. (**a**) Dataset A; (**b**) Dataset B; (**c**) Dataset C; (**d**) Dataset D.

To evaluate the reconstruction performance of the 3DGS algorithm under different scenarios and conditions, this study adopts 4 multimodal datasets, covering diverse scenes from indoor to outdoor facilities, and constructs a multi-dimensional test benchmark through different acquisition strategies and data characteristics. The detailed information of their data parameters is as follows (Table 1):

The scenes of the four types of experimental data are shown in Figure 4:

Dataset A captures a historic brick-built fortress in Shuozhou City, Shanxi Province, which is part of the ancient Great Wall, representing a significant cultural heritage site. Constructed from densely stacked masonry units, the structure exhibits complex surface geometry—including repetitive brick patterns, deep occlusions, and narrow gaps—posing challenges for 3D reconstruction. Data was collected using a DJI Matrice 4E drone with close-range photogrammetry to preserve fine texture and geometric detail. The SfM-generated sparse point cloud contains 794,699 points, well-distributed across façades and corridors. This dataset evaluates reconstruction performance on intricate, non-smooth rigid structures while supporting real-world applications in digital preservation and monitoring of historical architecture.

Dataset B focuses on medium-sized outdoor building structures in Guangzhou City, Guangdong Province, captured by a drone in a surrounding manner at a flying height of 100 m, taking into account both building facades and roof details. Its initial point cloud contains 116,281 sparse points, densely distributed in building outlines and surrounding open areas, suitable for evaluating the reconstruction accuracy and memory efficiency of the algorithm for large-scale rigid scenes.

Dataset C is aimed at water areas and natural landscape scenes in Xi’an City, Shaanxi Province. It was captured synchronously by a five-camera array mounted on a drone, covering the lake surface and shoreline according to a preset grid path to ensure three-dimensional coverage of dynamic water ripples and vegetation. Among the generated 54,723 initial point clouds, the point cloud in the water surface area is sparse due to reflective characteristics, while the point cloud in the land area is dense and contains vegetation topological structures. It focuses on testing the algorithm’s robustness to reflective surfaces and unstructured natural scenes.

Dataset D is oriented towards linear water conservancy facilities in Hainan Tibetan Autonomous Prefecture, Qinghai Province. A drone was used to perform an S-shaped snake-like flight along the flood embankment to cover the embankment horizontally. The initialized point cloud contains 91,984 sparse points, distributed linearly along the embankment with a significantly increased density at the slope protection structure. It aims to verify the algorithm’s global consistency optimization ability and computational efficiency for long-distance linear scenes.

The experiment utilized the open-source COLMAP framework for data preprocessing. Regarding the data partitioning strategy, a systematic sampling approach was adopted to divide input images into training and test sets at an 8:1 ratio. Specifically, one test sample was selected for every eight training images. This partitioning strategy ensures spatial independence between test and training perspectives, effectively preventing overfitting caused by viewpoint overlap in adjacent frames. Consequently, it objectively evaluates the model’s generalization capability and geometric consistency for unseen viewpoints.

### 3.2. Hardware Configuration

The 3DGS framework was implemented in Python 3.10, leveraging the PyTorch 2.1.0 deep learning framework with associated libraries (torchaudio 2.1.0, torchvision 0.20.1) for core algorithmic operations. Computational acceleration during training and inference was achieved through GPU-accelerated parallel processing via CUDA 11.8. Experiments were conducted on a high-performance computing server running Ubuntu 20.04 LTS (Kernel 5.15.0-78-generic), developed by Canonical (London, United Kingdom), to ensure system-level compatibility and stability. The hardware and software configurations of the experiment are shown in Table 2:

### 3.3. Efficiency Comparative Analysis

All computational tasks in this study were deployed on a headless high-performance computing (HPC) server cluster. Raw data was transferred to the server-side via secure remote protocols, with both data preprocessing and training procedures executed entirely on the servers. Upon training completion, the generated scene representation files (containing Gaussian parameters and pose data) were downloaded to local workstations for interactive 3D scene rendering and visual validation.

To comprehensively quantify the algorithm performance, on the basis of three image quality evaluation metrics (SSIM, PSNR, and LPIPS), the experiment further expanded the evaluation dimensions to computational efficiency and resource consumption. The training time, real-time rendering frame rate (FPS), and model memory usage of each dataset were recorded, constructing a multi-dimensional performance evaluation system.

We compare our approach against several state-of-the-art 3D reconstruction methods: Compact3D and GaussianPro, which represent recent advancements in efficient Gaussian splatting, as well as D^2^GS, a method that also leverages Depth-Anything V2 for metric depth initialization. All comparative experiments were conducted using the default configurations and released parameters provided by the respective authors to ensure a fair and reproducible evaluation. Specifically, D^2^GS employs the Depth-Anything V2 (Vitb) model for depth estimation, with no additional fine-tuning or parameter adjustments. The experimental results are as follows: 

Based on the comprehensive evaluation results in Table 3, the performance of existing 3D reconstruction methods varies significantly across datasets, underscoring their sensitivity to scene characteristics such as scale, geometric complexity, and surface properties. In contrast, our method demonstrates consistent superiority in both reconstruction quality and computational efficiency across all evaluated scenarios.

On Dataset A, our method achieves a test PSNR of 21.77 dB, outperforming GaussianPro (+4.61 dB) and D^2^GS (+10.71 dB), and is slightly lower than Compact3D (22.29 dB). It attains the highest SSIM (0.6363) and lowest LPIPS (0.4020), indicating superior structural and perceptual fidelity. Notably, our method reduces training time to 36 min 33 s—comparable to Compact3D (39 min 31 s)—while achieving a higher training PSNR (20.46 dB vs. 19.39 dB), suggesting better optimization stability. Although Compact3D achieves a higher FPS (183 vs. 102), our method maintains a reasonable speed-accuracy trade-off for detailed rigid scenes.

On Dataset B, our method achieves the best performance across all quality metrics: SSIM (0.9382), test PSNR (34.15 dB), and LPIPS (0.1095), surpassing all baselines. It also demonstrates superior efficiency, with a training time of only 17 min 12s—significantly faster than Compact3D (30 min 35 s) and GaussianPro (36 min 30 s)—and a high inference speed of 170 FPS, close to Compact3D (189 FPS) and much faster than others. With a memory of 0.28 GB, our method is highly efficient for medium-sized outdoor reconstruction, balancing speed, quality, and resource usage.

On Dataset C, which features challenging reflective water and vegetation, our method achieves the highest test PSNR (22.98 dB) and lowest LPIPS (0.2999), demonstrating strong generalization in complex natural environments. It operates at 71 FPS with 2.63 GB of memory, significantly outperforming GaussianPro (28 FPS, 3.25 GB) and D^2^GS (17 FPS, 0.81 GB) in speed and quality. Despite D2GS showing high training PSNR (28.44 dB), its low test PSNR (15.00 dB) indicates severe overfitting, whereas our method maintains balanced train/test performance (28.96 dB/22.98 dB), reflecting better generalization.

On Dataset D, our method achieves the highest SSIM (0.7352), test PSNR (19.51 dB), and lowest LPIPS (0.2936) among all compared methods, significantly outperforming others in perceptual and structural quality. Although its test PSNR is lower than on other datasets (likely due to sparse viewpoints and long-range structures), it still surpasses Compact3D (19.91 dB), GaussianPro (20.06 dB), and D^2^GS (13.12 dB) in LPIPS and SSIM. Training completes in 38m7s, and inference runs at 67 FPS with 2.98 GB memory—comparable to GaussianPro (4.39 GB) and acceptable given the improved output quality.

A key observation is the train-test PSNR gap, which reflects overfitting risk. D^2^GS and GaussianPro exhibit large gaps (e.g., D^2^GS on B: 36.60 vs. 20.26 dB), while our method maintains smaller gaps across datasets (e.g., on B: 35.24 vs. 34.15 dB), indicating better generalization. Additionally, our method achieves training PSNR > 28 dB on all datasets, confirming robust learning of observed views.

In summary, our approach consistently achieves top-tier reconstruction quality (SSIM, PSNR, LPIPS) across diverse scenes, with superior training efficiency and competitive runtime performance. It particularly excels in large-scale and complex natural environments, while maintaining robustness in challenging, sparse-view scenarios. These results validate the effectiveness of our gradient-aware dynamic adjustment framework in enhancing both accuracy and efficiency in 3DGS.

### 3.4. Comparative Analysis of the Impact of Resolution on Reconstruction Results

To address the training efficiency and memory pressure challenges faced by the 3DGS algorithm in large-scale complex scenes, this study further explores the optimization potential of input image resolution on reconstruction performance. Taking Dataset D as an example, training under its original resolution (4032 × 3024) required nearly 50 min with a memory footprint of 2.26 GB, highlighting the stringent demands of high-resolution data on computational resources. For this purpose, the experiment used the open-source tool ImageMagick [30] to perform batch downsampling on input images, generating three low-resolution datasets at 1/2, 1/4, and 1/8 of the original resolution, systematically analyzing the trade-off between reconstruction quality and computational efficiency when reducing resolution. The downsampling process employed the Lanczos filtering algorithm [31] to preserve primary geometric features while suppressing high-frequency noise, ensuring comparability across different resolution datasets.

**Figure 5:**
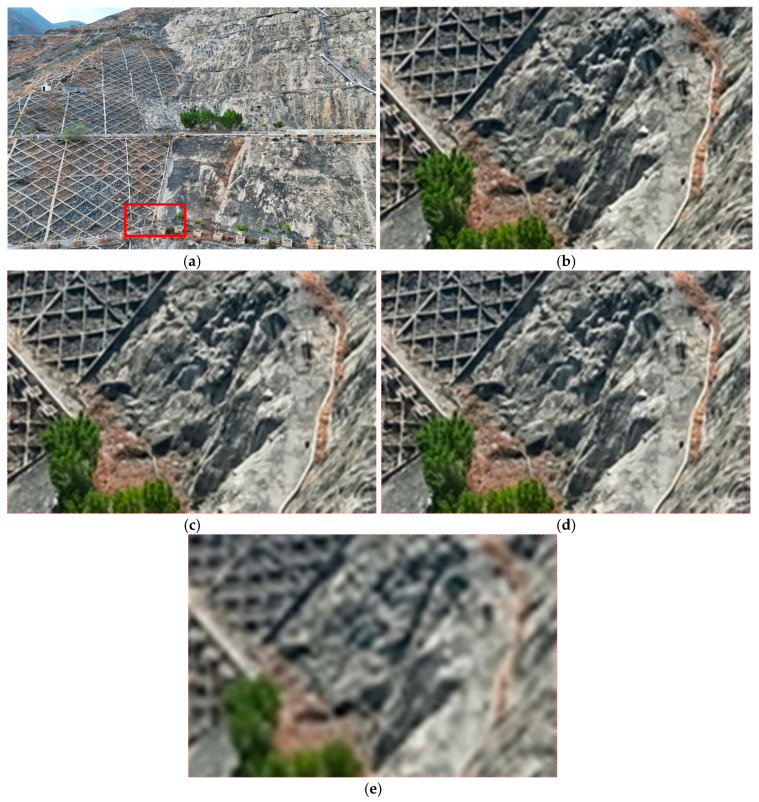
Reconstruction Results at Different Resolutions. (**a**) The red box represents the comparison area (**b**) Results at original resolution; (**c**) Results at 1/2 resolution; (**d**) Results at 1/4 resolution; (**e**) Results at 1/8 resolution.

The reconstruction results of Dataset D after downsampling are shown in Figure 5.

The accuracy statistics of the above experimental results are shown in Table 4.

A thorough analysis of how input resolution affects 3DGS reconstruction reveals a complex trade-off between quantitative performance, computational efficiency, and visual fidelity. While lower resolutions generally improve numerical metrics and training speed, they may simultaneously lead to the loss of fine geometric details.

On Dataset A, reducing resolution to 1/8 improves test PSNR from 21.77 dB to 23.76 dB, SSIM from 0.6363 to 0.6961, and reduces training time from 36 min 33 s to 5 min 39 s. This suggests that high-frequency noise in the input images may hinder optimization, and downsampling acts as a beneficial filter.

On Dataset B, medium-sized outdoor building structures, the benefits are even more pronounced: at 1/8 resolution, test PSNR increases from 34.15 dB to 36.90 dB, LPIPS drops to 0.0312, and training time is reduced from 17 min 12 s to 4 min 54 s. The rich texture and dense viewpoints make this scene highly resilient to resolution reduction.

On Dataset C, featuring reflective water and vegetation, test PSNR improves from 22.98 dB to 25.55 dB at 1/8 resolution, with LPIPS decreasing from 0.2999 to 0.1206 and memory usage dropping from 2.63 GB to 0.23 GB. The improved metrics suggest better consistency, though fine dynamic details may be smoothed.

On Dataset D, a long-range linear structure captured under sparse viewpoints, the quantitative gains from downsampling are substantial. At 1/8 resolution, test PSNR increases from 19.51 dB to 25.22 dB, SSIM improves from 0.7352 to 0.8480, and LPIPS is nearly halved. Training time drops from 38 min 7 s to 11 min 8 s, and memory usage is reduced from 2.98 GB to 0.75 GB—making high-quality reconstruction feasible on lower-end hardware.

However, visual inspection of the reconstruction results (Figure 5) reveals a critical trade-off. While the global structure and alignment are significantly improved—with fewer ghosting artifacts and better coherence in distant regions—fine geometric details are visibly degraded. Specifically, the grid structure of the embankment becomes fragmented and blurred, texture edges are noticeably softened, and small-scale topological features such as railings and surface patterns are smoothed out or lost. Moreover, the breaking of linear continuity further contributes to an overall “low-frequency approximation” of the original scene, where high-fidelity structural details are sacrificed despite improved quantitative metrics.

This contradiction arises from the dual effect of downsampling: while Lanczos filtering suppresses high-frequency noise and view-inconsistent artifacts that plague sparse-view reconstruction, excessive smoothing irreversibly removes critical edge and texture information essential for high-fidelity rendering. As a result, although macroscopic metrics are inflated, the practical visual quality in high-detail regions is diminished.

### 3.5. Impact of Different Depth Models on Results

In the 3DGS algorithm, the introduction of a depth prediction network aims to enhance the accuracy and robustness of few-sample reconstruction through geometric priors. This study focuses on three pre-trained models provided by the Depth-Anything V2 framework. Based on the medium-sized outdoor building structures (Dataset B), by comparing the performance metrics of the scenario without depth supervision (None) and those with the three models, it explores the impact of depth models of different specifications on the final reconstruction performance, and derives the trade-off relationship between model specification selection and the final reconstruction effect. The specific experimental results are described below. The quantitative results, summarized in Table 5, reveal a significant impact of the depth encoder on both reconstruction quality and system performance. In the absence of depth supervision (None), the baseline achieves an SSIM of 0.9233, a test PSNR of 33.84 dB, and an LPIPS of 0.1692, with highly efficient training (25 min 21 s) and rendering (220 FPS) at a memory cost of only 0.18 GB. This establishes a strong benchmark for fast and lightweight reconstruction.

When incorporating depth priors, the lightweight Vits model reduces training time to 17 min 15 s and maintains a moderate memory footprint (0.26 GB), but yields notably inferior visual quality, with SSIM dropping to 0.9023 and test PSNR decreasing to 31.35 dB. Although LPIPS improves slightly (0.1172), this may stem from over-smoothing rather than enhanced detail, as visual inspection reveals blurred textures and weakened geometric sharpness. The model’s limited capacity likely leads to underfitting, failing to capture complex depth structures necessary for high-fidelity reconstruction.

In contrast, the Vitb model achieves the best overall performance among depth-supervised configurations, delivering the highest SSIM (0.9382), the highest test PSNR (34.15 dB), and the lowest LPIPS (0.1095), indicating superior structural and perceptual quality. Despite a modest increase in memory usage (0.28 GB), it maintains competitive training and inference speeds (17m 12s, 170 FPS). This suggests that Vitb’s deeper feature hierarchy provides accurate and well-calibrated depth estimates that effectively enhance geometric coherence without introducing significant domain bias or destabilizing the optimization process.

Surprisingly, the largest model, Vitl, despite having the most parameters and the fastest training time (17 min 06 s), performs worst in terms of structural metrics, with SSIM dropping to 0.8996. While its test PSNR remains high (34.10 dB), the increase in LPIPS (0.1154) and qualitative assessment indicate over-smoothing and loss of fine-grained texture detail. The reduced training duration and final model fidelity suggest premature convergence, where strong semantic priors from the large pre-trained model dominate the depth prediction, leading to inaccurate geometric initialization and suboptimal Gaussian distribution fitting under limited input views.

These findings challenge the common assumption that larger pre-trained models inherently improve downstream 3D reconstruction. Instead, they highlight a nuanced relationship between model scale and performance: while the Vits model lacks sufficient representational power for complex scenes, the Vitl model suffers from over-regularization due to semantic bias, particularly in few-shot settings. The Vitb model, by contrast, achieves an optimal balance—its moderate depth and width enable effective adaptation to scene-specific geometry while preserving the ability to capture fine-scale details.

To further validate the quality of the depth priors, we compare the metric depth maps from Depth-Anything V2 against COLMAP’s sparse depth estimates across four datasets, as shown in Table 6.

On Dataset A, all three models produce similar RMSE values (5.114–5.122 m), indicating consistent depth estimation when scene complexity is low. However, on Datasets B, C, and D—characterized by greater depth range, texture scarcity, or structural complexity—both Vitb and Vitl significantly outperform Vits, achieving lower RMSE (e.g., 2.598 m vs. 2.711 m on B, 3.177 m vs. 3.211 m on C, and 5.314 m vs. 5.367 m on D). This confirms that larger models generate more accurate depth maps under challenging conditions, benefiting from stronger global context modeling and higher-resolution feature extraction.

Nevertheless, improved depth accuracy does not always translate to better rendering quality in 3DGS. While Vitl produces the most metrically accurate depth, its excessive reliance on high-level semantics appears to interfere with the delicate balance required for Gaussian optimization, ultimately degrading visual fidelity. In contrast, Vitb provides sufficiently accurate depth supervision—consistently outperforming Vits—while remaining adaptable and stable during training.

In summary, the experimental results demonstrate that the choice of depth encoder must balance representational power with task-specific adaptability. The lightweight Vits is insufficient for complex scenes, while the over-parameterized Vitl introduces detrimental biases despite accurate depth estimation. The Vitb model emerges as the most suitable choice, offering the best compromise between depth accuracy, geometric faithfulness, and reconstruction stability, thereby enabling high-quality 3DGS under few-view conditions.

### 3.6. Comparison of Ablation Analysis Results for the Proposed Method

To systematically evaluate the contribution of three core introduced modules—depth regularization, gradient dynamic adjustment, and floating noise detection and removal—to the performance of the improved algorithm, this study designs a progressive ablation experiment based on the standard 3DGS framework. By constructing 6 groups of control experiments, the activation states (1 for enabled, 0 for ablated) of depth supervision, densification condition triggering, and floating noise detection are controlled, respectively, to comprehensively quantify the influence weight of each module on geometric modeling and rendering quality.

All experiments were executed under identical hardware environments and hyperparameter configurations, using the indoor plush toy scene for training. Evaluation integrated three metrics—PSNR, SSIM, and LPIPS—to eliminate single-metric bias. This experimental design isolates variable effects through independent module combinations, thereby enabling granular analysis of each component’s specific impact.

The detailed results are presented below.

As shown in Table 7, when only depth regularization is enabled, the SSIM increases from the baseline of 0.9558 to 0.9621, and the test set PSNR rises from 31.70 dB to 32.42 dB, verifying the effectiveness of the depth prior in geometric constraints. However, the training time increases from 13 min 54 s to 32 min 03 s, reflecting the high computational load of depth supervision. When the gradient dynamic adjustment module is enabled independently, the improvement in metrics is limited (SSIM 0.9563, PSNR 31.71 dB), but its training time only increases by 11 s. This indicates that the module improves optimization stability with almost no additional resource consumption by optimizing the densification triggering logic.

The independent activation of the floating noise detection module leads to a significant decline in quality, with the test set PSNR dropping to 27.54 dB. This phenomenon may be attributed to the mismatch between the currently manually set fixed parameters and the scene characteristics of detection radius (0.04) and adjacent point threshold (3). For instance, in regions with sparse yet valid structures—such as foliage—low local density causes legitimate Gaussians to be misclassified as noise and prematurely removed. When all three modules are enabled together, although the PSNR increases to 32.87 dB, the LPIPS deteriorates to 0.3166. This suggests that modules such as depth regularization and densification, which mainly function in the later stage of modeling, are limited by the lack of early geometric information, resulting in incomplete generation of high-frequency details. Meanwhile, the manual threshold strategy also struggles to accurately distinguish between initial noise and potentially valid structures, highlighting the limitations of the fixed detection window. Finally, the synergistic effect of depth regularization and gradient dynamic adjustment shows a better balanced performance: the SSIM increases to 0.9665, and the training time is reduced by 38% compared with single depth supervision, shortening to 19 min 48 s. This indicates that gradient dynamic adjustment significantly alleviates the computational redundancy of depth supervision by adaptively triggering densification.

The experimental results show that depth regularization is the core driver of quality improvement, gradient dynamic adjustment enhances its universality through efficiency optimization, while the floating noise detection still needs further optimization in the design of the collaborative scheme for time window and threshold.

## 4. Conclusions and Discussion

This study conducts a systematic exploration of the 3DGS technology. Aiming at the bottlenecks in efficiency and accuracy of 3DGS in real-scene 3D modeling, it proposes a series of innovative optimization strategies. By introducing a depth prediction network, a gradient dynamic adjustment mechanism, and a floating noise detection method, the geometric reconstruction quality and algorithm robustness in few-sample scenarios are significantly improved. The enhanced framework achieves robust performance in medium-scale outdoor scenes (e.g., SSIM = 0.9382, PSNR = 34.15 dB on Dataset B with 170+ FPS at 1600 × 900 resolution), effectively rendering complex materials like dynamic water ripples and layered vegetation via explicit Gaussian representations. However, scalability challenges emerge in large-area modeling (e.g., 2.98 GB memory, 40 min training for Dataset D, PSNR = 19.51 dB), highlighting hardware dependency. Excessive input downsampling causes high-frequency detail loss, underscoring sensitivity to data quality. Depth model selection also critically impacts performance: Vitb achieves competitive results with lower overhead, while Vitl introduces domain bias in certain scenes, suggesting the need for scene-aware adaptation.

To address these limitations, we plan to integrate advanced Gaussian compression techniques (e.g., Compact3D, LightGaussian) to balance model compactness and rendering quality. Additionally, we aim to redesign the density-based pruning module with adaptive thresholds that respond dynamically to local Gaussian density and gradient saliency, enabling smarter suppression of floating artifacts while preserving structurally and visually salient features.

In general, 3DGS technology achieves a breakthrough balance between real-time rendering and geometric fidelity through the differentiable optimization of explicit Gaussian ellipsoids and efficient rasterization pipelines, providing a new technical path for application scenarios such as cultural heritage protection and smart city construction. However, its challenges in modeling weak texture areas, adapting to dynamic scenes, and hardware resource consumption still need to be further addressed.

## Figures and Tables

**Table 1:** Detailed Parameters of the Image Dataset.

Dataset	Sensor	Manufacturer	Country	Number of Images	Image Size (Pixel)	SfM Points
A	DJI Matrice 4E	DJI Innovations	China	51	1600 × 1160	44,550
B	—	—	—	127	1600 × 900	116,281
C	SONY ILCE-6000	Sony Corporation	China	125	1600 × 1067	54,723
D	DJI M3TD	DJI Innovations	China	101	1600 × 1200	91,984

**Table 2:** Experimental Hardware and Software Configuration.

Category	Component	Specifications
Hardware	CPU	Intel Xeon Platinum 8352V 16 vCPUs 2.10 GHz base/3.50 GHz Turbo Boost
	Memory	90 GB DDR5-3200 ECC RAM
	GPU	NVIDIA GeForce RTX 4090 24 GB GDDR6X VRAM 16,384 CUDA cores 2.52 GHz boost clock FP32/FP16 mixed-precision RT Core acceleration
Software	Drivers	NVIDIA Driver 560.35.03
	Compute Platform	CUDA Toolkit 11.8
	Core Library	Custom diff-gaussian-rasterization Differentiable rasterization pipeline Gaussian splat projection Gradient backpropagation
	Environment Management	Miniconda 25.9.1 Dependency isolation Reproducibility assurance

**Table 3:** Training Results of Each Dataset (↑: improvement; ↓: degradation).

Method	Dataset	SSIM ↑	PSNR (Test) ↑ (dB)	PSNR (Train) ↑ (dB)	LPIPS ↓	Train ↓	FPS ↑	Memory ↓
Compact 3D	A	0.6217	22.2858	19.3870	0.4532	39 min 31 s	183	0.19 GB
	B	0.9219	33.8784	32.8203	0.1401	30 min 35 s	189	0.17 GB
	C	0.7138	21.9022	30.2133	0.3036	87 min 49 s	122	0.85 GB
	D	0.5324	19.9144	21.9899	0.4173	36 min 31 s	69	1.74 GB
GaussianPro	A	0.5686	17.1633	23.1615	0.4588	49 min 48 s	92	0.91 GB
	B	0.9300	33.4725	35.1082	0.1238	36 min 30 s	134	0.52 GB
	C	0.6840	22.2534	31.0061	0.3230	58 min 09 s	28	3.25 GB
	D	0.5765	20.0643	23.7567	0.3360	47 min 25 s	20	4.39 GB
D^2^GS	A	0.2497	11.0559	26.1732	0.6902	40 min 57 s	12	1.84 GB
	B	0.6209	20.2605	36.5967	0.4498	26 min 30 s	19	0.78 GB
	C	0.4566	14.9969	28.4386	0.6744	34 min 07 s	17	0.81 GB
	D	0.2164	13.1219	25.4361	0.6529	40 min 30 s	14	1.00 GB
Ours	A	0.6363	21.7713	20.4556	0.4020	36 min 33 s	102	1.56 GB
	B	0.9382	34.1519	35.2433	0.1095	17 min 12 s	170	0.28 GB
	C	0.6899	22.9760	28.9641	0.2999	21 min 14 s	71	2.63 GB
	D	0.7352	19.5129	23.5860	0.2936	38 min 07 s	67	2.98 GB

**Table 4:** Training Results at Different Resolutions (↑: improvement; ↓: degradation).

Dataset	Data Type	SSIM ↑	PSNR (Test) ↑ (dB)	PSNR (Train) ↑ (dB)	LPIPS ↓	Train ↓	FPS ↑	Memory ↓
A	D 1	0.6363	21.7713	20.4556	0.4020	36 min 33 s	102	1.56 GB
	D 1/2	0.6701	22.2980	24.9616	0.3901	14 min 34 s	145	0.69 GB
	D 1/4	0.6899	23.2715	29.3054	0.3574	8 min 37 s	192	0.21 GB
	D 1/8	0.6961	23.7621	23.7672	0.3380	5 min 39 s	240	0.18 GB
B	D 1	0.9382	34.1519	35.2433	0.1095	17 min 12 s	170	0.28 GB
	D 1/2	0.9273	34.7998	36.1036	0.1066	8 min 35 s	208	0.20 GB
	D 1/4	0.9545	35.7703	37.3308	0.0558	6 min 01 s	220	0.16 GB
	D 1/8	0.9730	36.8994	38.8639	0.0312	4 min 54 s	248	0.06 GB
C	D 1	0.6899	22.9760	28.9641	0.2999	21 min 14 s	71	2.63 GB
	D 1/2	0.7679	24.3359	31.7485	0.2050	17 min 18 s	90	1.84 GB
	D 1/4	0.7801	25.4567	32.7515	0.1463	9 min 27 s	136	0.83 GB
	D 1/8	0.7853	25.5544	33.4214	0.1206	5 min 54 s	189	0.23 GB
D	D 1	0.7352	19.5129	23.5860	0.2936	38 min 07 s	67	2.98 GB
	D 1/2	0.8003	20.6186	26.7363	0.2808	29 min 06 s	80	2.19 GB
	D 1/4	0.7723	23.0365	29.5460	0.19126	22 min 44 s	95	1.64 GB
	D 1/8	0.8480	25.2150	31.7621	0.1400	11 min 08 s	140	0.75 GB

**Table 5:** Impact of Different Depth Models on Training Results (↑: improvement; ↓: degradation).

Data Type	SSIM ↑	PSNR (Test) ↑ (dB)	PSNR (Train) ↑ (dB)	LPIPS ↓	Train ↓	FPS ↑	Memory ↓
None	0.9233	33.8404	35.2824	0.1692	25 min 21 s	220	0.18 GB
Vits	0.9023	31.3508	32.4879	0.1172	17 min 15 s	172	0.26 GB
Vitb	0.9382	34.1519	35.2433	0.1095	17 min 12 s	170	0.28 GB
Vitl	0.8996	34.1018	35.1750	0.1154	17 min 06 s	168	0.34 GB

**Table 6:** Accuracy of Depth-Anything V2 compared with COLMAP.

Dataset	A			B			C			D		
Data type	Vits	Vitb	Vitl	Vits	Vitb	Vitl	Vits	Vitb	Vitl	Vits	Vitb	Vitl
RMSE (m)	5.114	5.117	5.122	2.711	2.628	2.598	3.211	3.180	3.177	5.367	5.318	5.314

**Table 7:** Comparative Analysis of Ablation Experiment Results (↑: improvement; ↓: degradation).

Depth	Gradient	Floating Points	SSIM ↑	PSNR (Test) ↑ (dB)	PSNR (Train) ↑ (dB)	LPIPS ↓	Train ↓
0	0	0	0.9558	31.7016	37.5429	0.2028	13 min 54 s
1	0	0	0.9621	32.4223	37.6367	0.2016	32 min 3 s
0	1	0	0.9563	31.7074	37.5705	0.2057	14 min 5 s
0	0	1	0.8206	27.5357	31.3648	0.4191	18 min 20 s
1	1	0	0.9665	32.6397	37.8967	0.2033	19 min 48 s
1	1	1	0.9198	32.8730	37.9829	0.3166	19 min 9 s

## Data Availability

The data presented in this study are available on request from the corresponding author.
